# Effects of Exogenous Recombinant APC in Mouse Models of Ischemia Reperfusion Injury and of Atherosclerosis

**DOI:** 10.1371/journal.pone.0101446

**Published:** 2014-07-17

**Authors:** Karin C. A. A. Wildhagen, Roy Schrijver, Linda Beckers, Hugo ten Cate, Chris P. M. Reutelingsperger, Esther Lutgens, Gerry A. F. Nicolaes

**Affiliations:** 1 Department of Biochemistry, Cardiovascular Research Institute Maastricht, Maastricht University, Maastricht, The Netherlands; 2 Department of Medical Biochemistry, Academic Medical Center, Amsterdam, The Netherlands; 3 Department of Internal Medicine, Laboratory for Clinical Thrombosis and Haemostasis, Cardiovascular Research Institute Maastricht, Maastricht University, Maastricht, The Netherlands; Institut National de la Santé et de la Recherche Médicale, France

## Abstract

Activated protein C (APC) is a serine protease that has both anticoagulant and cytoprotective properties. The cytoprotective effects are protease activated receptor 1 (PAR-1) and endothelial protein C receptor (EPCR) dependent and likely underlie protective effects of APC in animal models of sepsis, myocardial infarction and ischemic stroke. S360A-(A)PC, a variant (A)PC that has no catalytic activity, binds EPCR and shifts pro-inflammatory signaling of the thrombin-PAR-1 complex to anti-inflammatory signaling. In this study we investigated effects of human (h)wt-PC, hS360A-PC, hwt-APC and hS360A-APC in acute (mouse model of acute myocardial ischemia/reperfusion (I/R) injury) and chronic inflammation (apoE^−/−^ mouse model of atherosclerosis). All h(A)PC variants significantly reduced myocardial infarct area (p<0.05) following I/R injury. IL-6 levels in heart homogenates did not differ significantly between sham, placebo and treatment groups in I/R injury. None of the h(A)PC variants decreased number and size of atherosclerotic plaques in apoE^−/−^ mice. Only hS360A-APC slightly affected phenotype of plaques. IL-6 levels in plasma were significantly (p<0.001) decreased in hwt-APC and hS360A-PC treated mice. In the last group levels of monocyte chemotactic protein 1 (MCP-1) were significantly increased (p<0.05). In this study we show that both hwt and hS360A-(A)PC protect against acute myocardial I/R injury, which implies that protection from I/R injury is independent of the proteolytic activity of APC. However, in the chronic atherosclerosis model hwt and hS360-(A)PC had only minor effects. When the dose, species and mode of (A)PC administration will be adjusted, we believe that (A)PC will have potential to influence development of chronic inflammation as occurring during atherosclerosis as well.

## Introduction

Protein C (PC) circulates as an inactive zymogen that can be activated by thrombin bound to the endothelium associated transmembrane receptor thrombomodulin. When PC is bound to the endothelial protein C receptor (EPCR), its activation is enhanced by 20-fold [1]. Activated protein C (APC) is a serine protease that, together with its cofactors protein S and factor V (FV), regulates thrombin formation by proteolysis of the active coagulation factors V (FVa) and VIII (FVIIIa) [2]–[5]. The anticoagulant PC pathway is vital to normal haemostasis, as indicated by the increased risk for thromboembolic events in heterogeneous PC deficiency [6], [7] and the life threatening thrombotic complications observed, even in the presence of aggressive anticoagulant therapy, in homozygous PC deficiency [8], [9].

APC has initially been identified and characterized as an anticoagulant protein; however recent interest has focused more on the cytoprotective functions of APC. APC has anti-inflammatory, anti-apoptotic and endothelial barrier stabilizing effects in different cell types, that are protease activated receptor 1 (PAR-1) and EPCR-dependent [10], [11]. These cellular effects, rather than the anticoagulant activity of APC, likely explain the protective effects of APC observed in animal studies of sepsis, lung injury, myocardial infarction and ischemic stroke [12]–[16].

The active site of APC contains the conserved catalytic triad: residues H211, D257 and S360. Mutation of the active site serine to alanine (S360A) yields a protein that is not able to cleave substrates, but still retains some anticoagulant activity through its ability to compete with activated coagulation factors X and IX for binding to FVa and FVIIIa, respectively [17], [18]. The cell surface receptor PAR-1 is involved in mediation of the cell-protective properties of APC. S360A-APC, however fails to cleave PAR-1 and is therefore regarded as being devoid of cytoprotective effects [19]. Thrombin is also able to cleave PAR-1 and in contrast to APC cleaved PAR-1, thrombin cleaved PAR-1 causes permeability-enhancing and pro-inflammatory effects via G_q_ and G_12/13_ mediated signaling [20]. Interestingly, it has been shown that binding of APC, PC as well as S360A-PC to EPCR, is associated with PAR-1 coupling to a G(i/o) family of G-proteins, resulting in initiation of protective signaling after cleavage of PAR-1 by either APC or thrombin [21]. Thus, this implies that protective PAR-1 signaling can also be initiated by PC and by proteolytically inactive S360A-(A)PC. Therefore we hypothesize that not only human wild type (hwt)-APC, but also hwt-PC, hS360A-PC and hS360A-APC may initiate cytoprotective signaling *in vivo* via EPCR-dependent modulation of PAR-1 signaling.

In the present study we have investigated the cytoprotective effects of human hwt-PC, hS360A-PC, hwt-APC and hS360A-APC in two different cardiovascular relevant mouse models: myocardial ischemia/reperfusion (I/R) injury and atherosclerosis. hAPC is very similar to murine (m) APC (69% sequence identity) [22]. It is known that mAPC is significantly more potent than hAPC as an anticoagulant in mice [15], but we hypothesize that anticoagulation is not necessary for protection in our models. Previous studies have shown that hAPC has positive effects on lesion volume, inflammation, vascular permeability and neurological function in murine models studying I/R injury and stroke [23]–[27].

Inflammation plays a key role in the pathogenesis of both myocardial I/R injury and atherosclerosis models in the current study, although the sequence of immune cell infiltration and the characteristics of the immune cell infiltrate are markedly different. After acute myocardial I/R, neutrophils migrate into the infarct zone within 24 hours, where they release granular enzymes and reactive oxygen species and cause vascular plugging [28]. In atherosclerosis, mainly T-cells and monocytes are recruited into the plaque, where monocytes mature into macrophages and finally foam cells. These immune cells release cytokines within the plaque and promote the expression of matrix metalloproteinases that break down collagen and thereby make plaques more vulnerable [29]. We hypothesize that hwt-(A)PC and hS360A-(A)PC will protect against acute inflammation in the myocardial I/R injury model and chronic inflammation in the atherosclerosis model.

## Materials & Methods

### Expression and purification of recombinant human (S360A)-PC

Recombinant hS360A-PC was obtained by PCR-based site-directed mutagenesis of hwt-PC cDNA. The cDNA of hwt-PC and hS360A-PC were inserted into the eukaryotic expression vector pRc/CMV (Invitrogen), transfected into HEK293 cells (CRL-1573 ATCC) and recombinant h(S360A)-PC was purified and characterized as described earlier [30], [31]. The purity and integrity of h(S360A)-PC was evaluated by SDS-PAGE (Figure S1). h(S360A)-PC concentrations were quantified by measurement of absorbance at 280 nm using an absorption coefficient of 14.5 (280 nm, 1%, 1cm) and by ELISA, employing the horse anti-human PC polyclonal antibody PAHPC-H (HTI), as a catcher antibody and the horseradish peroxidase-conjugated anti-human PC polyclonal Dako P0374 as a detecting antibody [18].

### Activation of h(S360A)-PC and catalytic activity against small substrates

hwt-PC and hS360A-PC were activated with a 1 ml HiTrap NHS-Activated HP column (GE Healthcare) to which 50 Units of carrier-free Protac (Pentapharm) had prior been conjugated. Up to 0.5 mg of h(S360A)-PC was incubated for 2 hours on the column at room temperature, and was next eluted and concentrated with a 10 K Macrosep Advance Centrifugal Device (Pall Life Sciences). Complete activation of the hwt-PC was ascertained by measuring the amidolytic activity of APC with S-2366 (Chromogenix) directly and after 60 minutes incubation in the presence of additionally added (0.05 units/ml final concentration) Protac. We confirmed the absence of Protac from the APC preps generated. For both hwt- and hS360A-PC, completion of activation was verified by SDS PAGE analysis (Figure S1). APC concentrations were determined from the above-described PC ELISA.

### Mice

All animal experiments were approved by the Animal Ethics Committee of Maastricht University. All mice were housed and bred according to institutional guidelines and were fed a normal chow diet. For the myocardial I/R experiment male C57BL/6 mice (Charles River Laboratories) of 8 weeks old and 25 g were used (n = 13 per group). For the atherosclerosis experiments, 21-week old male ApoE^−/−^ mice were used (Charles River Laboratories/in house breeding facility Maastricht University). Treatment, twice weekly, with intraperitoneal (i.p.) injections of saline (n = 20) or 0.2 mg/kg of hwt-PC (n = 13), hS360A-PC (n = 12), hwt-APC (n = 16) or hS360A-APC (n = 16) started at week 21 and was continued for 8 weeks. The 0.2 mg/kg dose has been proven protective in models of myocardial I/R injury [23], hepatic I/R injury [25] and stroke [15], [32], [33].

### Procedure myocardial I/R

Myocardial I/R was performed as described earlier [34]. In short, mice were anaesthetized with isoflurane and were ventilated during the ischemic period. Ischemia was induced by ligation of the left anterior descending (LAD) artery for 60 minutes followed by 2 hours of reperfusion. hwt-PC, hS360A-PC, hwt-APC or hS360A-APC (0.4 mg/kg) or placebo (saline) were administered intravenously (i.v.) both 15 minutes after induction of ischemia and 5 minutes after induction of reperfusion. After reperfusion, the animals were anaesthetized as described earlier. Heart tissue was collected for determination of infarct size (n = 6 per group) or interleukin (IL)-6 levels (n = 7 per group).

### Myocardial I/R: determination of infarct size

Infarct size was determined by Evans Blue/triphenyl tetrazolium chloride (TTC) staining [35]. In brief, after reperfusion, the LAD was re-ligated and Evans Blue dye was injected i.v. and was allowed to circulate for 2 min to identify the area at risk (AAR). After excision of the heart, it was cut into 0.5-mm-thick slices and TTC was added to mark the viable cells within the AAR red. The non-viable cells remained pale and defined the area of infarction (AOI). Pictures of the slices were taken and were analyzed by Adobe Photoshop CS2 to determine infarct sizes (AOI/AAR ratios).

### Myocardial I/R: determination of IL-6 levels

Hearts of mice were excised, lyophilized and the resulting dry heart powder was dissolved in glucopyranoside. After determination of protein levels with DC Protein Assay Kit II (Bio-Rad), tissue homogenates were diluted to 5 mg/ml protein and IL-6 levels per mg heart homogenate were determined with mouse IL-6 ELISA Ready-set-go (eBiosciences).

### Atherosclerosis: tissue processing, histology, and morphometry

At the end of the experimental period, at 29 weeks of age, mice were anaesthetized with pentobarbital (100 mg/kg, i.p.). Blood was taken in EDTA via cardiac puncture, followed by perfusion of the arterial tree under physiological pressure with PBS containing sodium nitroprusside (Sigma-Aldrich) and subsequently by 1% paraformaldehyde. The aortic arch including its main branch points was removed, fixed overnight in 1% paraformaldehyde, longitudinally embedded in paraffin, and sectioned. Twenty consecutive sections (4 µm) representing the central area of the aortic arch with an intact morphology of the arch and branch points were selected. For histological analysis of atherosclerosis, 4 sections (20 µm apart) were stained with hematoxylin and eosin (H&E) and the atherosclerotic lesions were classified as “initial” or “advanced” according to the Virmani classification [36]. Plaque area was determined using a Leica DM3000 light microscope and a 10/0.3 NA on 20/0.5 objective (Leica Microsystems) coupled to a computerized morphometry system (Qwin 3.5; Leica, Wetzlar, Germany). Images were captured using a Leica DFC 425c camera. Morphometric parameters were determined as described previously [37].

### Atherosclerosis: flow cytometry

Blood and spleen were collected, processed and fluorescently stained with either antibody mix 1 (CD3-FITC, CD4-PerCp, CD8-eFLUO450, CD25-APC, Foxp3-PE and B220-PE-Cy7) or antibody mix 2 (CD11b-PE-Cy7, Ly6C-APC, Ly6G-PE, CD3-FITC, B220-APC-eFLUO780 and NK1.1-PerCp-Cy5.5) or isotype control IgG (all BD Biosciences or eBioscience). Cells were analysed using a FACS-Canto II (BD Biosciences) flowcytometer.

### Atherosclerosis: (immuno)histochemistry

Aortic arch sections were immunolabeled with anti-Mac3 monoclonal antibody (mAb) (1∶30; BD Biosciences PharMingen) to detect macrophages, anti-CD3 (1∶200; Dako) to detect T lymphocytes and anti-CD45 mAb (1∶5000; BD Biosciences) to detect leukocytes. Antibody staining was visualized by diaminobenzidine or Vectastain red. Sirius red staining was performed for analysis of collagen content.

### Atherosclerosis: analysis of cytokine and cholesterol levels

Enzyme-linked immunosorbent assays (ELISAs) were used to quantify concentrations of cytokines IL-6, IL-10 and MCP-1 (all R&D systems) in plasma. Cholesterol levels in plasma were measured using the Cholesterol CHOD-PAP method (Roche).

### Statistical analysis

Data analysis was performed with GraphPad Prism 5. Data were presented as mean +/− SEM. Differences between groups were tested using One-way ANOVA and Dunnett’s multiple comparison post hoc test. P<0.05 was considered statistically significant.

## Results

### Expression and purification of recombinant h(S360A)-PC

hwt-PC and hS360A-PC were expressed under serum-free conditions at levels of 5 mg/liter and purified with overall recoveries of ∼40%. SDS-PAGE gel analysis under reducing conditions, revealed the presence of the light chain as well as the α, β and γ isoforms of the heavy chain (Figure S1). Upon activation by Protac, a fast-acting PC activator isolated from the venom of the copperhead snake *Agkistrodon contortrix*, the heavy chain isoforms shifted to a slightly lower migration position, consistent with full activation (95%) as judged by SDS-PAGE (Figure S1). The purified hS360A-APC preparation contained no measurable (<0.03%) amidolytic activity toward the chromogenic substrate S-2366.

### hwt- and hS360A-(A)PC reduce infarct area in a myocardial I/R mouse model

**Figure 1:**
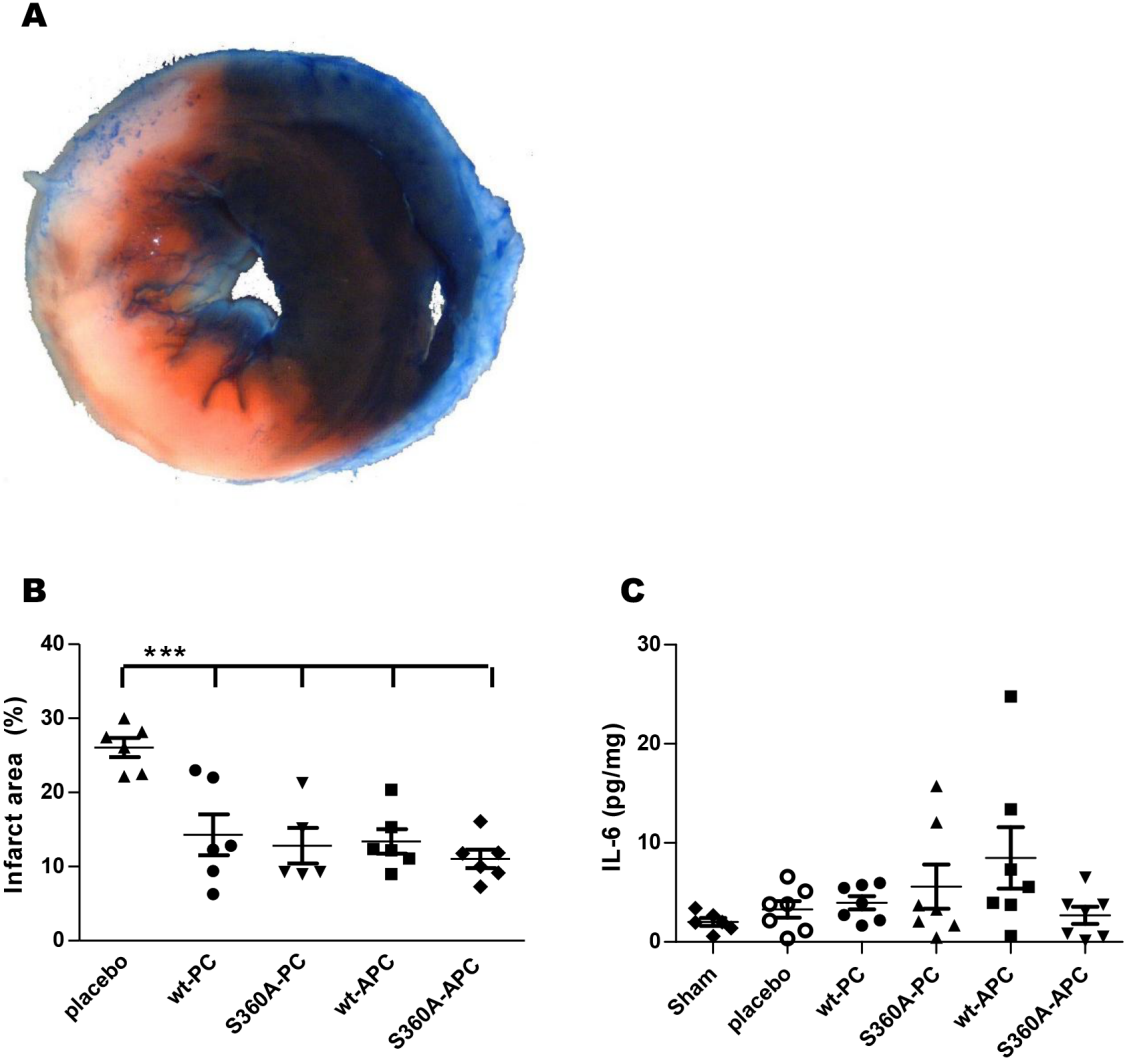
Treatment with wt- or S360A-(A)PC decreases infarct area. Mice were subjected to 1(0.4 mg/kg) or placebo (saline) were administered i.v. 15 min after induction of ischemia and a second time 5 minutes after induction of reperfusion. **A)** Representative picture of the Evans Blue/TTC staining after 2 h reperfusion. The normal tissue (non infarcted area) is stained dark blue, whereas the ischemic area (area at risk (AAR)) is stained brick red and the non-viable infarct area (area of infarction (AOI)) did not stain and remained pale. **B)** Infarct area (percentage AOI of AAR) as determined by Evans Blue/TTC staining. **C)** IL-6 concentrations were determined in heart tissue homogenates (5 mg/ml protein content) by ELISA and IL-6 per mg heart homogenate was calculated. All graphs show mean +/− SEM, statistical significance was tested using one-way analysis of variance with Dunnett post hoc test, ***P<0.001.

To examine the effects of APC treatment on myocardial I/R injury, mice were subjected to 1 hour ischemia by ligation of a suture around the LAD artery, followed by 2 hours of reperfusion. Mice received a first dose (0.4 mg/kg) of hwt-PC, hS360A-PC, hwt-APC, hS360A-APC or placebo (saline) 15 minutes after induction of ischemia and a second dose of the same treatment 5 minutes after induction of reperfusion. Next, infarct areas in the different groups were determined by Evans Blue/TTC staining (Figure 1A). Figure 1B shows that the infarct area in the placebo-treated mice was 26.1%. Remarkably, hwt-PC, hS360A-PC, hwt-APC and hS360A-APC treatment all significantly reduced myocardial infarct area (P<0.05) to values of 11.1% to 14.3%.

### hwt- and hS360A-(A)PC have no effect on IL-6 levels in a myocardial I/R mouse model

Interleukin 6 (IL-6) is a cytokine with pro-inflammatory and pro-coagulant properties that stimulates hepatocytes to produce several acute-phase proteins, like C reactive protein. Increased levels of IL-6 were found in coronary heart disease patients and were associated with prognosis [38]. We measured IL-6 levels in tissue homogenates of heart tissue from mice that underwent myocardial I/R. We observed no significant difference in IL-6 levels between mice that received placebo and mice in the different h(A)PC treatment groups (Figure 1C).

### hwt- and hS360A-(A)PC do not influence plaque area in ApoE^−/−^ mice

**Figure 2:**
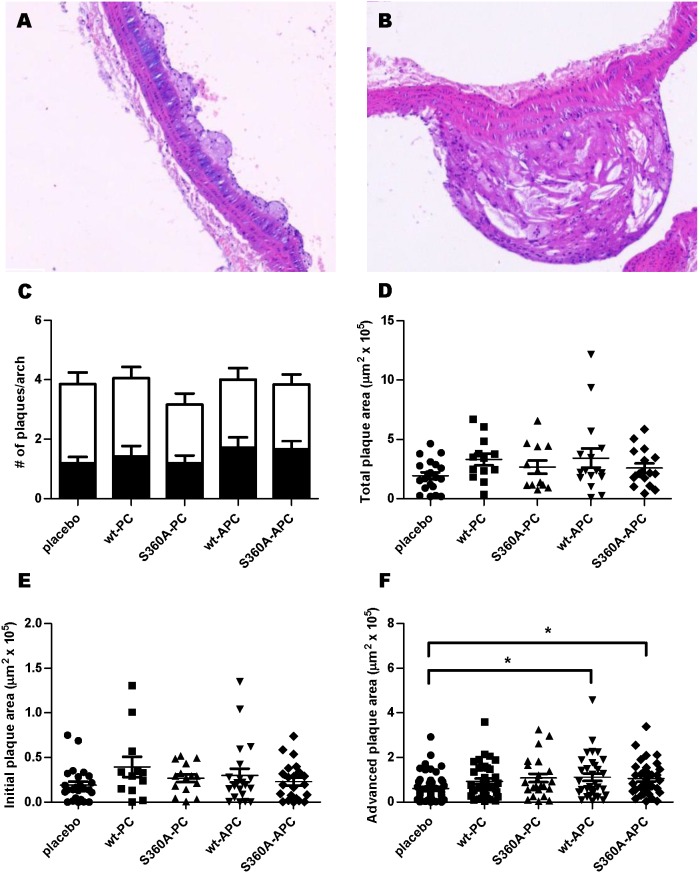
Treatment with wt- or S360A-(A)PC does not alter plaque area or # of plaques. ApoE^−/−^ mice were injected with saline or 0.2 mg/kg of wt-PC, S360A-PC, wt-APC or S360A-APC. Images of an initial (**A**) and an advanced (**B**) plaque after hematoxylin and eosin (H&E) staining. **C)** Atherosclerotic lesions were classified as initial (black bars) or advanced (white bars) and numbers of both type of plaques per mouse were determined. **D)** Total plaque area in the aortic arch as determined by H&E staining. **E)** Surface area of individual initial plaques. **F)** Surface area of individual advanced plaques. All graphs show mean +/− SEM, statistical significance was tested using one-way analysis of variance with Dunnett post hoc test, *P<0.05.

Having observed that all hPC and hAPC forms conferred cytoprotection in the acute inflammatory myocardial I/R model, we subsequently investigated the effect of hAPC administration on plaque development in a chronic inflammatory mouse model of atherosclerosis. ApoE^−/−^ mice were treated twice weekly with placebo, or hwt-PC, hS360A-PC, hwt-APC or hS360A-APC (0.2 mg/kg) for 8 weeks, after which atherosclerotic plaque development in the aortic arch was determined. Representative images of hematoxylin and eosin (H&E) stained initial and advanced plaques are shown in Figure 2A–B. On average, we detected 1 initial plaque and 3 advanced plaques in the mice from all groups analyzed (Figure 2C). We did observe a (non-significant) reduction in the total number of plaques for the hS360A-PC group. The number of advanced plaques was greatest in the placebo group and slightly decreased in the groups receiving treatment. Surprisingly, in the four treatment groups, total plaque area was not significantly different from the placebo group (Figure 2D). The areas of the individual initial plaques were comparable between groups (Figure 2E), with the placebo, hS360A-PC and hS360A-APC groups presenting with the lowest inter-individual variation. Surprisingly, the areas of individual advanced plaques were significantly increased in the hwt-APC and hS360A-APC groups compared to the placebo group (Figure 2F), although the data points of plaque sizes in the five different groups show considerable overlap. So, overall no clear effect of hwt- or hS360A-(A)PC on plaque development was observed.

### Effect of h(A)PC variants on plaque phenotype and the immune system

**Figure 3:**
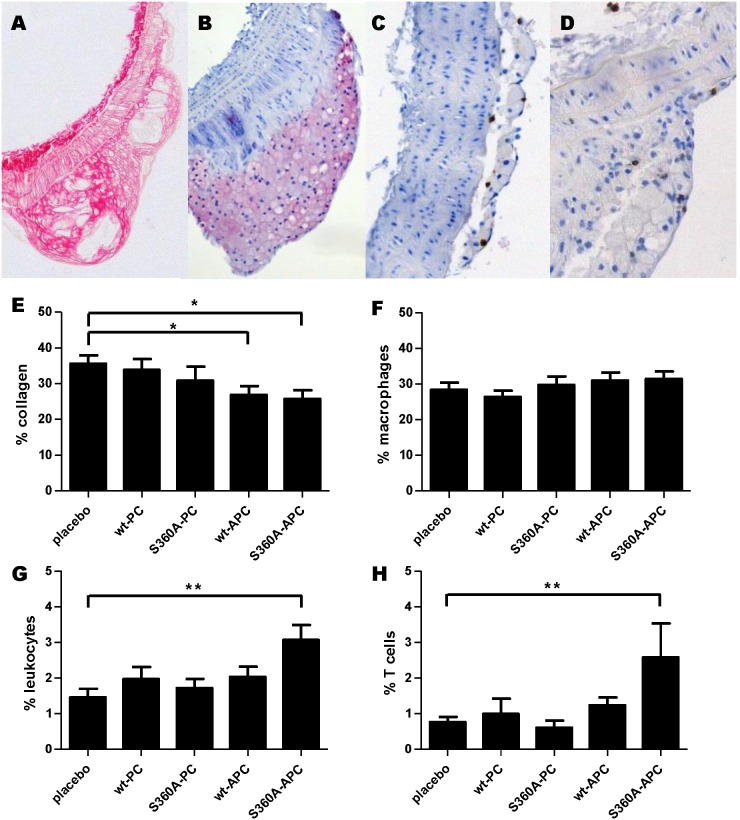
Only S360A-APC treatment influences plaque phenotype. Images of plaques stained for collagen (**A**), macrophages (**B**), leukocytes (**C**), and T cells (**D**). **E)** After sirius red staining, the percentage of collagen was determined by dividing the area of collagen by the total plaque area. **F)** After Mac-3 staining, the percentage of macrophages as a percentage of the total number of cells per plaque was calculated. **G)** After CD45 staining, the percentage of leukocytes as a percentage of the total number of cells per plaque was calculated. **H)** After CD3 staining, the percentage of T cells of the total number of cells per plaque was calculated. All graphs show mean +/− SEM, statistical significance was tested using one-way analysis of variance with Dunnett post hoc test, *P<0.05, **P<0.01.

Subsequently the plaque phenotype was further characterized. Collagen content (Figure 3A/E) and infiltration of macrophages (Figure 3B/F), leukocytes (Figure 3C/G) and T cells (Figure 3D/H) were determined in all plaques. hS360A-APC treated mice had, compared to placebo treated mice, significantly lower collagen content in their plaques (P<0.05), while they had increased leukocyte (P<0.01) and T cell (P<0.01) infiltration. Interestingly, also the hwt-APC treated group presented with significantly lower collagen content in their plaques (P<0.05). In the hwt-PC and hwt-APC groups, we noted a trend towards increased T cell and leukocyte counts. Infiltration of macrophages in plaques had a low variation and was not different between the placebo and different treatment groups. So, the plaque phenotype was slightly changed by hS360A-APC treatment and not by other treatments.

The effects of hAPC treatment on the innate and adaptive immune system were assessed by flow cytometric analysis of immune cells isolated from both blood and spleen. No significant differences were observed between quantities of B cells, granulocytes, NK cells, NKT cells and different types of monocytes (Ly6C^high/l^°^w/−^) and T cells (CD4^+^, CD8^+^, CD25^+^ and Foxp3^+^) (Figure S2–S4).

### Effect of h(A)PC variants on plasma levels of cytokines IL-6, IL-10 and MCP-1

**Figure 4:**
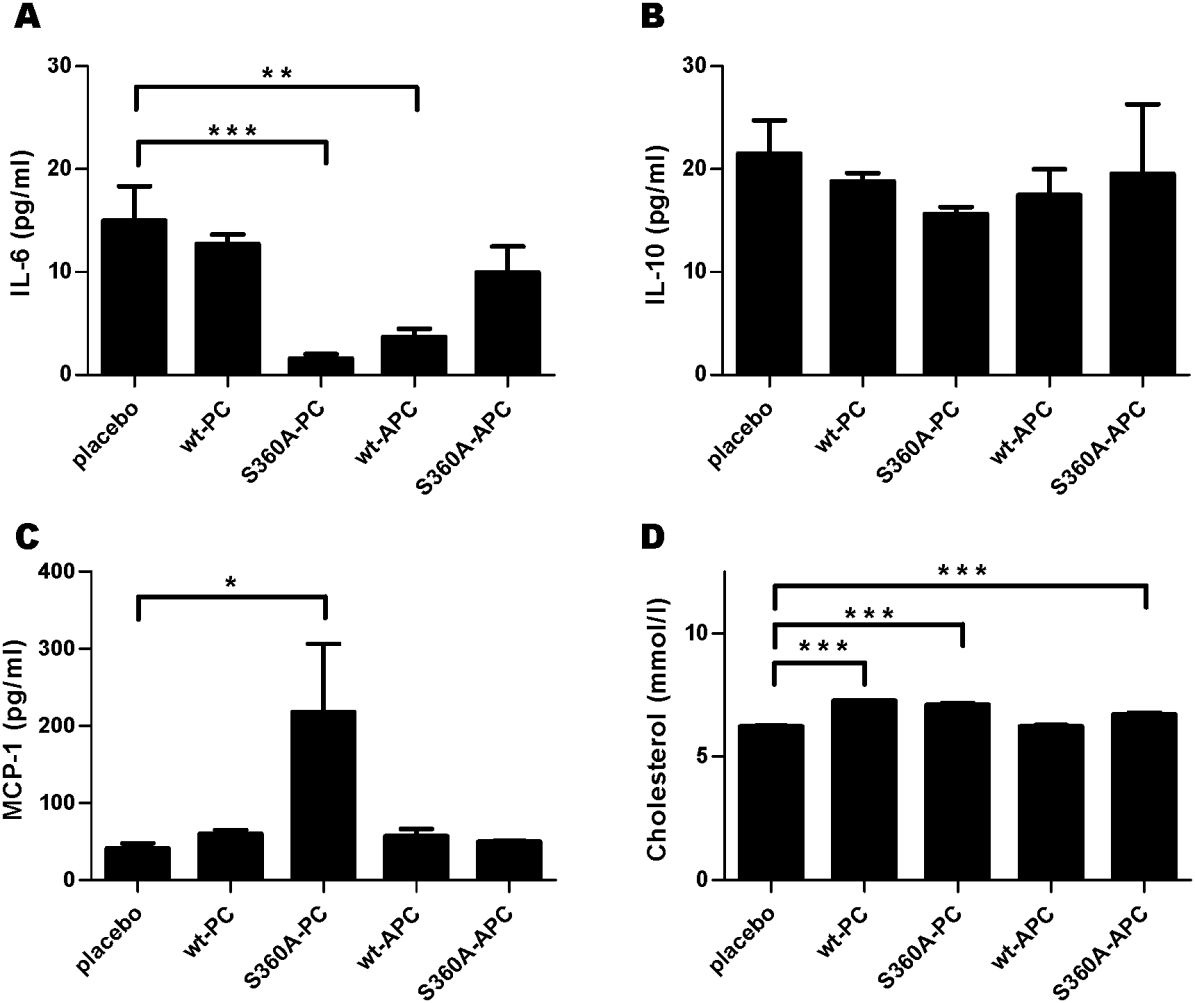
Effects of wt- and S360A-(A)PC treatment on cytokine and cholesterol levels. **A)** Interleukin (IL) 6 levels measured with ELISA in mouse plasma pools. **B)** IL-10 levels measured with ELISA in mouse plasma pools. **C)** Monocyte chemotactic protein-1 (MCP-1) levels measured with ELISA in mouse plasma pools. **D)** Cholesterol levels in mouse plasma pools measured with cholesterol CHOD-PAP method. All graphs show mean +/− SEM, statistical significance was tested using one-way analysis of variance with Dunnett post hoc test, *P<0.05, **P<0.01, ***P<0.001.

We next determined the levels of cytokines IL-6, IL-10 and MCP-1 and of cholesterol in the plasma from the animals included in this study and to this end prepared plasma pools by mixing equal volumes of plasma for each of the animals studied per group. Mice treated with hS360A-PC had significantly lower IL-6 levels (P<0.001) and increased MCP-1 levels (P<0.05), see Figure 4A/C. In the hwt-APC group, the IL-6 levels were also lower than in the placebo group (P<0.01). The latter group was highest of all groups determined here, for both the IL-6 and the IL-10 measurements. A non-significant trend was observed for the lowering effect of hS360A-PC on IL-10 levels (Figure 4B). Cholesterol levels were slightly but significantly higher in mice treated with hwt-PC, hS360A-PC and hS360A-APC compared to placebo treated mice (Figure 4D).

## Discussion

Several elegant studies have previously provided experimental evidence that wt-APC protects against acute myocardial I/R injury via both anti-inflammatory and anti-apoptotic effects (Table S1, refs [39]–[50]). In this study we show that not only hwt-APC’, but also hwt-PC’, hS360A-PC and hS360A-APC significantly reduce infarct area in a myocardial I/R model. This finding’, which is a phenomenological observation at this point’, deserves further mechanistic explanation. It has been shown [51]–[53] that EPCR and PAR-1 are associated with caveolin-1 within endothelial lipid rafts. When EPCR is bound to the gamma-carboxyglutamic (Gla)-domain of either wt-(A)PC or S360A-(A)PC, it dissociates from caveolin-1 and alters the G-protein mediated signaling of PAR-1 from pro-inflammatory (G_q_ and/or G_12/13_) to anti-inflammatory signaling (pertussis toxin–sensitive G_i_-protein). In this caveolin-unbound state, cleavage of PAR-1 by thrombin or APC and resulting G_i_ activation, initiates a barrier protective and anti-inflammatory response, which includes activation of Rac1 and inhibition of RhoA and NFκB signaling cascades. We speculate that these mechanisms likely contributed to the protective effects observed for the hPC and hAPC forms tested by us, including the proteolytically inactive hS360A-(A)PC variant. Despite the mutation of the active site, S360A-(A)PC is still able to bind EPCR via its Gla-domain [54], [55] and can thereby influence PAR-1 signaling pathways.

Additionally it was shown that both PC and APC significantly decrease LPS-induced reactive oxygen species (ROS) generation by macrophages. Remarkably these effects were EPCR-independent and only in APC-treated cells accompanied by inhibited NFκB activation [56]. Alternatively, these antioxidant effects of (A)PC may be related to inhibition of NADPH oxidase or by regulation of transcription factor Sp1 [57]. PC and APC reduced copper-induced LDL peroxidation via interaction of their Gla-domains with phospholipids on the surface of LDL particles, and protected unsaturated fatty acids against oxidation [56]. Both increased ROS generation and lipid peroxidation are observed during I/R injury and have been described to play an important role in the pathophysiology of myocardial injury [58], [59]. The observation that also zymogen PC provides *in vitro* antioxidant activity implies that the antioxidant properties are at least partly independent of the catalytic side and in line with our finding that both hwt- and hS360A-(A)PC have protective properties in acute myocardial I/R injury. There are several possible explanations for the observation that zymogen PC induced a similar reduction in infarct size as APC, while in fact its antioxidant capacities are lower than those of APC [56]. Firstly, activation of PC on endothelial cells generates APC with 4-fold higher cytoprotective barrier protective properties than exogenous APC [54]. This discrepancy cannot be explained by differences in the local APC concentrations and possibly originates because protective signaling by APC is mechanistically linked to PC activation [54]. Additionally, both zymogen PC and S360A-(A)PC have the advantage that these are not inhibited by circulating serpins and therefore have a longer half-life *in vivo* [17], which would make these proteins better suited for potential application in a clinical therapeutic context, like for organ/tissue protection. Additional beneficial properties can be attributed to S360A-PC, since this molecule, unlike its wild-type homolog, is unlikely to result in bleeding side effects after *in vivo* thrombomodulin-mediated activation by thrombin.

Although S360A-(A)PC still has residual anticoagulant activity because of its ability to limit thrombin formation through competition with FXa and FIXa for resp. FVa and FVIIIa, it is unlikely that these anticoagulant properties of S360A-(A)PC have contributed to the reduced myocardial infarct area observed in S360A-(A)PC treated mice. It has been shown before [14], [60] that the use of *in vivo* thrombin inhibition by heparin administration or the selective thrombin generation inhibitor dansyl glutamyl-glycyl-arginyl chloromethyl ketone-treated activated factor X (DEGR-FXa), failed to reduce myocardial I/R injury and I/R-induced spinal cord injury. Likewise, another study by Wang and coworkers [61] compared the effects of the cytoprotective-selective 5A-APC variant (<10% anticoagulant but normal cytoprotective activity) and the anticoagulant-selective E149A-APC variant (>3-fold increased anticoagulant activity but defective cytoprotective activities) in a murine focal ischemic stroke model. Despite its reduced anticoagulant activity, 5A-APC significantly decreased infarct- and edema volume and improved neurological outcome, while E149A-APC administration resulted in significantly worsened neurological outcome and increased infarct- and edema volume. Additionally, E149A-APC treatment was associated with an increased risk of bleeding as indicated by 5-fold increased hemoglobin levels in the ischemic brain.

In contrast to the study of Loubele [14] and coworkers we did not find a significant effect of h(A)PC treatment on IL-6 levels in heart homogenates after acute myocardial I/R injury. One possible explanation for the difference is the fact that IL-6 levels in placebo treated mice were 5-fold lower in the present study, leaving less room for a further decrease by h(A)PC treatment. The discrepancy may also arise from the fact that we used h(A)PC and Loubele and co-workers mAPC. Previous research has shown that hAPC was significantly less potent in murine stroke models as compared to mAPC [24]. The observed difference cannot be explained by a difference in proteolytic activity, but the lower affinity of hAPC for mPAR-1 than for the human isoform of this receptor probably plays a role [62]. Differences in sequence (69% sequence identity between hAPC and mAPC [22]) or post-translational modifications can possibly explain these different affinities.

While we hypothesized that administration of h(A)PC would also influence plaque development in the long term chronic atherosclerosis mouse model, we found that none of the h(A)PC variants significantly protected against the development of atherosclerosis in this mouse model of chronic inflammation. hwt-APC and hS360A-APC even slightly increased plaque area of advanced plaques, but the differences between these groups and the other groups were relatively small. Probably the lack of effect of h(A)PC on reducing plaque development can be explained by the *in vivo* bioavailability after injection. Previously it was shown that i.p. administration of 0.1 or 0.8 mg/kg mAPC produces a transient rise in APC levels in circulation for 3 hours, with a plateau after around 20 min [32], [63]. This transient rise in (A)PC concentration in circulation, that was achieved twice a week in our model, was apparently not enough to initiate protective signaling that lasted long enough to provide long term protection against atherosclerosis development during the time span of our study.

We have however observed a clear effect of hS360A-PC and hwt-APC administration on IL-6 levels in plasma. The decrease in concentration of the pro-inflammatory cytokine IL-6 after hS360A-PC and hwt-APC treatment can possibly be explained by inhibited IL-6 release by neutrophils as a result of h(A)PC treatment [64]. Conflicting reports on the role of IL-6 in the development of murine atherosclerosis have appeared; some studies show that IL-6 promotes atherosclerosis while other studies show that IL-6^−/−^ mice are more atherogenic than control mice, so it is not clear what the exact contribution of IL-6 to atherogenesis is [65]–[70]. In hS360A-PC treated mice we found an increased level of MCP-1. Remarkably, this was not accompanied by increased percentages of leukocytes and macrophages in plaques and therefore likely had no effects on plaque development. In hS360A-APC treated mice the phenotype of the atherosclerotic plaques was slightly changed. Plaques contained significantly less collagen, while the infiltration of both leukocytes and T-cells was increased. Both an increase in activated immune cells and a decrease of collagen in plaques are associated with a more unstable plaque phenotype [71], [72].

Analysis of other cardiovascular studies which have studied the *in vivo* effects of (A)PC in a cardiovascular context (Table S1) reveals that administration of APC has mainly been studied in models of acute diseases like I/R injury and stroke. In most of these experimental models administration of APC improved primary and secondary outcome variables, like survival, lesion/edema volume, cardiac/neurological function and lowered the concentration of inflammation markers. For comparison between experiments it is important to realize that administration of 0.2 mg/kg APC i.v. and 0.8 mg/kg APC i.p. result in similar APC plasma pharmacokinetic profiles [32]. Wang et al. [23] show that an APC mutant that is not able to cleave PAR-1 (E170A-PC) does not have any protective effect, while an APC mutant with greatly impaired anticoagulant function (3K3A-APC) was as effective as wt-APC in decreasing lesion volume and improving neurological function. This result in itself suggests that mainly the cytoprotective PAR-1-dependent effects of APC, rather than the anticoagulant functions of APC, are important for protection in cardiovascular disease. However, the current study showed that hS360A-APC was as effective as hwt-APC in decreasing lesion volume in myocardial I/R injury, although this variant had strongly decreased anti-coagulant activity and was not able to cleave PAR-1. We hypothesize that hS360A-APC was protective because it can bind through its Gla-domain to EPCR and thereby promotes protective PAR-1 signaling via endogenous mAPC and thrombin [21]. Both these serine proteases are known to be formed during I/R damage [73], [74]. Alternatively, hS360A APC may reduce ROS formation and bind through its Gla-domain to phospholipids on LDL particles, resulting in reduced lipid peroxidation as discussed above for PC and APC. Both effects can contribute to decreased lesion volume in acute myocardial I/R injury.

We have studied the effects of 4 different h(A)PC variants in mouse models for cardiovascular disease: in an acute I/R injury model and in a chronic atherosclerosis model. We have shown that treatment with both hwt and hS360A-(A)PC decreases infarct area in our acute murine myocardial I/R injury model. However, no significant effects were observed for the development of atherosclerotic plaques, a chronic slow process. Trends were observed that indicate that h(A)PC may be able to reduce in particular IL-6 and collagen levels, but under the conditions used in our experiments these did not lead to significant differences in total plaque area or plaque phenotype. Comparison of our data with those in literature learns that the (A)PC dose, mode of administration and species of administered (A)PC varied among different studies. We therefore propose that for future experiments in chronic disease models using mice, it will be preferable to include at least mAPC, which is administered preferably intravenously and more frequently than in the current study. If possible a slow-release device would be a preferred method, but this will require that the (A)PC preparations are stable for extended periods of time at body temperature. Nonetheless, while recognizing that such controlled study has not been provided yet, we regard our current study as valuable information that will enable the needed further study. In conclusion, this study showed that both hwt and hS360A-(A)PC protect in an acute murine myocardial I/R injury model and have the potential to influence development of chronic inflammation as occurring during atherosclerosis as well.

## Supporting Information

Figure S1 **SDS page gel of human wt- and S360A-(A)PC.** Reduced samples of human wt-PC (lane 1), wt-APC (lane 2), S360A-PC (lane 3) and S360A-APC (lane 4) were loaded on a 10% SDS PAGE gel and afterwards stained with Coomassie Brilliant Blue.
(TIF)

Figure S2 **Effect of wt- and S360A-(A)PC treatment on immune cells in blood.** Percentages of **A)** NK cells (NK1–1+, CD3–), **B)** NKT cells (NK1–1+, CD3+), **C)** Granulocytes (CD11b+, Ly6G+) and **D)** monocytes (CD11b+, Ly6G−) were determined by flow cytometric analysis of mice blood. **E)** Monocytes were further characterized as Ly6C high (♦), Ly6C low (♦) or Ly6C− (◊). Graphs A–D show mean +/− SEM.
(TIF)

Figure S3 **Effect of wt- and S360A-(A)PC treatment on immune cells in blood.** Percentages of **A)** total T cells (CD3+), **B)** CD8+ T cells, **C)** CD4+ T cells, **D)** regulatory T cells (FOXP3+, CD25+) and **E)** B cells (B220+) were determined by flow cytometric analysis of mice blood. All graphs show mean +/− SEM.
(TIF)

Figure S4 **Effect of wt- and S360A-(A)PC treatment on immune cells isolated from spleen.** Percentages of **A)** total T cells (CD3+), **B)** CD8+ T cells, **C)** CD4+ T cells, **D)** regulatory T cells (FOXP3+, CD25+) and **E)** B cells (B220+) were determined by flow cytometric analysis of cells isolated from mice spleen tissue. All graphs show mean +/− SEM, statistical significance was tested using one-way analysis of variance with Dunnett post hoc test, *P<0.05.
(TIF)

Table S1 Overview experimental APC studies in cardiovascular diseases. Overview of studies that used different models to study effects of (A)PC on cardiovascular diseases. For each study the species, APC type, dose and injection method and effects on outcome measurements were shown. Abbreviations: ALT: alanine aminotransferase; APC: activated protein C; BBB: blood brain barrier; BCAO: bilateral carotid artery occlusion; CBF: cerebral blood flow; ERK: Extracellular signal-regulated protein kinases; h: human; IL: interleukin; ip: intraperitoneal; I/R: ischemia/reperfusion; iv: intravenous; JNK: c-Jun N-terminal kinase; m: mouse; MAP: mean arterial pressure; MCAO: middle cerebral artery occlusion; MMP: matrix metalloproteinase; MOD: multiple organ dysfunction; MPO: myeloperoxidase; NFκB: nuclear factor kappa B; pd: plasma derived; r: recombinant; TNFα: tumor necrosis factor α; wt: wild type.
(TIF)
