# Correction: Effects of Exogenous Recombinant APC in Mouse Models of Ischemia Reperfusion Injury and of Atherosclerosis

**DOI:** 10.1371/journal.pone.0109498

**Published:** 2014-09-26

**Authors:** The PLOS ONE Staff

The affiliation for the sixth author is incorrect. Esther Lutgens is also affiliated with: Institute for Cardiovascular Prevention, Ludwig-Maximilians-University Munich, Munich Germany.
