# Supplementary figures and images for: Effects of Exogenous Recombinant APC in Mouse Models of Ischemia Reperfusion Injury and of Atherosclerosis
Source: PLoS One. 2014 Jul 17;9(7):e101446. doi: 10.1371/journal.pone.0101446 (PMC4102480; doi:10.1371/journal.pone.0101446)

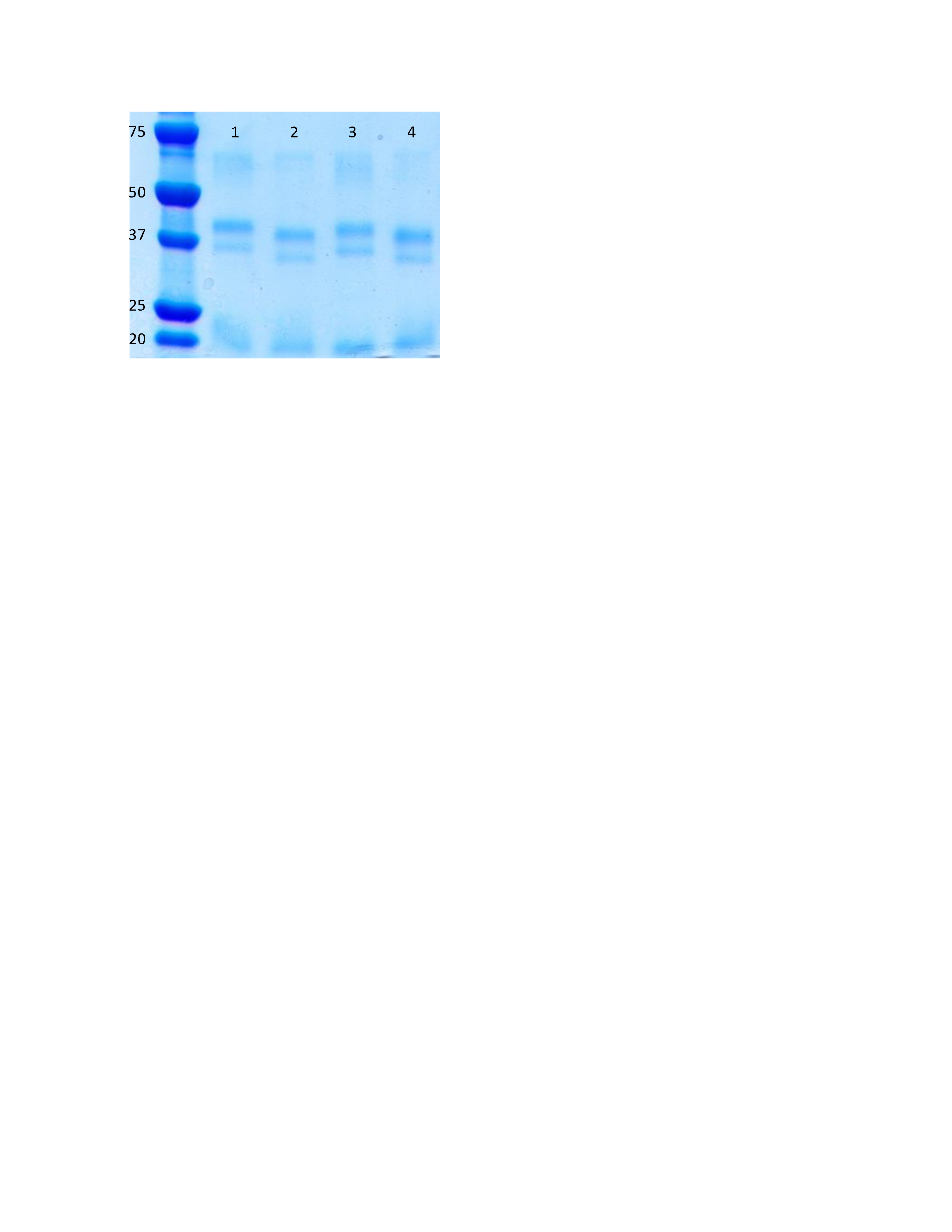

Supplement: Figure S1 — SDS page gel of human wt- and S360A-(A)PC. Reduced samples of human wt-PC (lane 1), wt-APC (lane 2), S360A-PC (lane 3) and S360A-APC (lane 4) were loaded on a 10% SDS PAGE gel and afterwards stained with Coomassie Brilliant Blue. (TIF) [file pone.0101446.s001.tif]

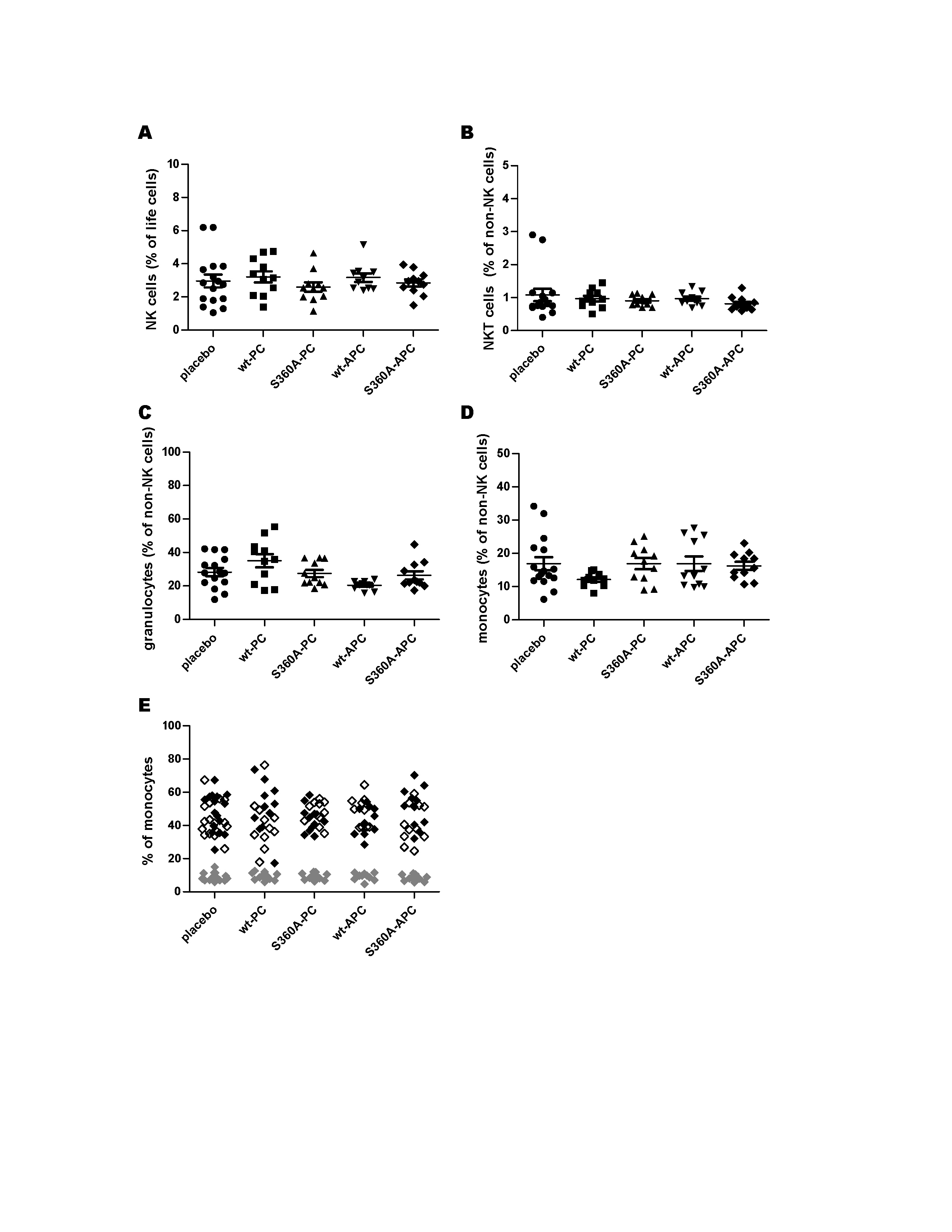

Supplement: Figure S2 — Effect of wt- and S360A-(A)PC treatment on immune cells in blood. Percentages of A) NK cells (NK1–1+, CD3–), B) NKT cells (NK1–1+, CD3+), C) Granulocytes (CD11b+, Ly6G+) and D) monocytes (CD11b+, Ly6G−) were determined by flow cytometric analysis of mice blood. E) Monocytes were further characterized as Ly6C high (♦), Ly6C low (♦) or Ly6C− (◊). Graphs A–D show mean +/− SEM. (TIF) [file pone.0101446.s002.tif]
